# Dynamic Operability Analysis for Process Design and Control of Modular Natural Gas Utilization Systems

**DOI:** 10.1021/acs.iecr.2c03543

**Published:** 2023-01-17

**Authors:** San Dinh, Fernando V. Lima

**Affiliations:** Department of Chemical and Biomedical Engineering, West Virginia University, Morgantown, West Virginia26506, United States

## Abstract

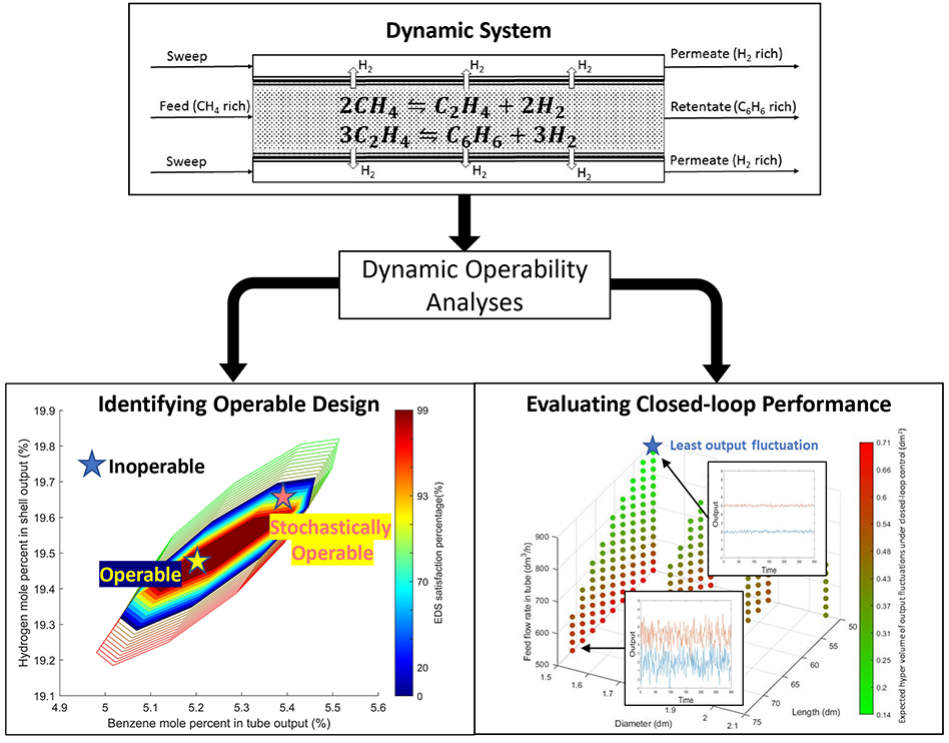

Process modularization is an alternative process design and construction framework, in which modular units are independent and replaceable blocks of a process system. While modular plants have higher efficiency and are safer to construct than conventional stick-built plants (Roy, S. *Chem. Eng. Prog*. **2017**, *113*, 28–31), they are significantly more challenging to operate because of the loss in the control degrees of freedom that comes with process integration and intensification (Bishop, B. A.; Lima, F. V. *Processes***2021**, *9*, 2165). To address this challenge, in this work, operability analyses are performed to consider the design and operation of modular units. Initially, a steady-state operability analysis is employed to find a set of feasible modular designs that are able to operate considering different modular plant conditions. A dynamic operability analysis is then applied to the feasible designs to identify the operable designs that are capable of rejecting the operational disturbances. Lastly, a closed-loop control measure is introduced to compare the performances of the different operable designs. The proposed approach is implemented in a modular membrane reactor to find a set of operable designs considering different natural gas wells, and the respective closed-loop nonlinear model predictive control performance of these units is evaluated.

## 1 Introduction

Process modularization is an emerging process design framework that provides an alternative to the typical stick-built construction approach for chemical processes. In this framework, individual modular units are a series of standardized units that are fabricated in manufacturing facilities, and then installed and replaced based on the needs of the production site to construct a modular plant. Since modular units are assembled in a factory, higher quality control is achieved, and worker safety is increased.^3^ Additionally, modularization of a process can potentially save on capital cost, deployment cost, and project timeline.^4−6^ In recent years, process intensification has been combined with modularization resulting in many significant improvements in process design.^2,7^ Through process intensification, new process designs with favorable scaling characteristics can be created as a means of further expanding modular manufacturing. Because an intensified modular process incorporates different phenomena (e.g., reaction, separation, heat transfer) into a single piece of equipment (e.g., membrane reactor, reactive distillation column), it is typically more efficient than its conventional counterparts.

While modularization and intensification provide the aforementioned advantages, they bring up a unique set of challenges. In traditional chemical plants, the process design is oversized to increase operability under uncertainties, but the design of a modular process aims to be more compact, which may cause the process to reduce its operable regions.^8^ Furthermore, modular plants are more difficult to control because of the coupling of phenomena that occurs during process intensification. This coupling of phenomena is the reason for greater efficiency in modular intensified processes, but the trade-off is fewer degrees of freedom for the controller because there are physically fewer manipulable streams and control valves in the intensified process when compared to the stand-alone units.^2^ Lastly, the dimensions of a modular unit are constrained by its means of transportation. For instance, when processing natural gas from the Marcellus Shale Formation around West Virginia and Pennsylvania, modular units can be shipped by road, resulting in their dimensions not exceeding those of commercial trucks.^9^ To address these challenges, a comprehensive framework for the design and control of modular units under the effects of process disturbances is proposed in this article.

In the chemical process industry, integrated design and control were recognized as early as 1964,^10^ but their application remained scarce for the subsequent years.^11^ For this purpose, multiobjective optimization problems were formulated to incorporate controllability with the economic objective using a relative gain array, minimum singular value, and condition number measures with a steady-state model.^12^ A different approach was to perform system identification on the nonlinear first-principles model to arrive at a linear model with a model uncertainty component to represent the mismatch between the models. Robust control tools were also applied to calculate the bounds of process feasibility and controllability.^13^ The method of steady-state flexibility optimization was first proposed as a max-min-max constrained problem to systematically account for the uncertainty in the process design problem.^14^ This method was later extended to include a dynamic optimization with time-varying uncertainty.^15^ So far, the aforementioned approaches only considered the uncertainty at its worst-case scenario without gauging its probability distribution. One method to overcome this limitation was to formulate a multistage stochastic programming problem with a scenario tree to represent possible events at discretized uncertainty realizations.^16^ A different approach based on Monte Carlo simulation was proposed to sample the random parameters and solve the integrated design and control problem as a regular dynamic programming problem to achieve a local solution. In this approach, the local solution was updated to move toward the new local solution until the back-off was invariant.^17^

In the last decades, process operability has emerged as an approach to quantify the operating region of a certain design and the feasibility of reaching all set points within this region. Process operability concepts appear in many areas of process systems engineering, such as process resiliency and optimization-based flexibility, and each area has a unique definition of an operable process. Process resiliency research defines operability as a dynamic performance index, which is formulated as a mean squared error associated with a closed-loop control system.^18^ Thus, resiliency-based operability is dependent on the controller’s formulation and does not reveal the dynamic capability of a process design. In flexibility research, operability is defined as the ability to satisfy inequality process constraints during operation,^19^ and it is generalized as a combination of flexibility and risk assessment. In the context of simultaneous design and control, the input-output geometric operability concept^20^ is the most suitable for finding feasible designs and operations of modular systems, since it provides a meaningful quantification involving the inputs, outputs, and disturbances. Although the flexibility-based operability and the geometric operability concepts share the same objective of analyzing the ability to achieve desired output specifications under the presence of disturbances, flexibility-based operability uses a max-min-max constrained optimization to find the best performance under the worst-case scenario of the disturbances, while the geometric operability explores all possible scenarios of a process at every value of the disturbances. Furthermore, since the geometric operability concept is an extension of the controllability concept from modern control theory, operability in this case, and as used in this article, is an inherent characteristic of a dynamic system and thus independent of the control law. Additionally, the operability analysis formulated here can be adapted to include other advanced process control notions, such as null controllable regions^21,22^ and positive invariant sets,^23^ so that the proposed operability algorithms provide researchers a convenient tool to calculate, visualize, and study such novel sets.

A brief summary of the benchmark contributions in operability research along with a comprehensive overview of past efforts is provided in references (24) and (25). Additionally, high-dimensional steady-state operability approaches were demonstrated to be effective in the case study of the Tennessee Eastman process,^26,27^ but the concept was not generalized for different design and control analyses. In the area of process modularization, a parallelized nonlinear programming problem (NLP) was formulated to find the optimal points for intensification of energy systems within the feasible design region given by the operability analysis.^28^ However, such an NLP approach was shown to be computationally expensive, and a linearized multimodel approximation was proposed, in which a mixed-integer linear programming problem (MILP) is formulated to replace the NLP.^9^ The MILP-based algorithm demonstrated significantly reduced computational time, while still being able to find a solution that was an asymptote to the NLP solution when applied to a multilayer design and control framework. However, this MILP-based framework did not consider the process disturbances for the design analysis, and the process dynamics for the control analysis. Dynamic operability was previously defined considering the minimal time to reach a new steady-state as a measure.^29^ This concept was later extended to construct an output funnel for feasible output transient constraints of advanced controllers^30^ and time-varying output and disturbance sets for a batch process.^31^ While the existing works on dynamic operability all provide meaningful results for their respective applications, a generalized definition of dynamic operability that retains the original motivation as a controllability measure^32^ is yet to be introduced in the literature. To fill this gap, a unified dynamic operability concept is proposed in this article with two different adaptations to represent the complex relationships between the design, the control structure, and the control law of a given modular process.

The remaining sections of this paper are organized as follows: A brief review of steady-state operability and the extended definition of dynamic operability is provided in section 2. Then, the proposed design and control framework for modular units is discussed in section 3. In section 4, a case study of a direct methane aromatization membrane reactor (DMA-MR) is provided to demonstrate the proposed framework. Finally, the results are summarized in section 5 with conclusions and potential future research directions.

## 2 Process Operability Concepts

A process is operable if the desired steady-state and dynamic performance requirements can be achieved from the available inputs regardless of the realization of the disturbances.^20^ In order to perform the operability analysis, a mathematical model is required that relates the inputs and the disturbances to the outputs of a given process. When a first-principles-based model is not available, a data-driven model (for example machine learning-based^33^) can be used for the operability analysis. Because the operability sets in the following sections are defined from the inputs, outputs, and disturbances of any given process, the definitions in these sections still hold regardless of the nature of the employed mathematical model. In this work, a first-principles model is assumed to be available to describe the system behavior, in which the state-space representation is given by the following differential-algebraic system of equations:
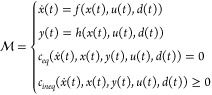
1in which at any given time *t*,  is the state vector,  is the manipulated/input vector,  is the disturbance vector, and  is the process output vector. The time derivative vector of the state is denoted as *ẋ*(*t*). The dynamics of the process are embedded in the rate of change equations . Also, the projections of the state vector onto the output vector correspond to the nonlinear mapping . The equality constraints and the inequality constraints are respectively *c*_*eq*_ and *c*_*ineq*_.

In the following sections, operability sets are introduced as means to quantify the relationships between the inputs and outputs of a process. The inputs of a process are often bounded above and below. For example, an irreversible flow input is bounded below by zero and bounded above by its equipment’s limitations. A measure, μ, is introduced as an effective means to compare two sets in the same space:
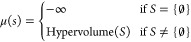
2in which the set  can either be an input set, an output set, or a disturbance set. The measure μ of the set *S* is an extension of the hypervolume, which is also the Lebesgue measure, in the Euclidean space containing *S*. If *S* is an interval in , μ measures its length. If *S* is a compact set in , μ measures its area. If *S* is a compact set in , μ measures its volume. If *S* is a compact set in a higher dimension, μ measures its hypervolume. When μ(*S*) is zero, *S* is not empty, but the set contains an infinitesimal subset of its enclosing space. For instance, the measure of a line segment in a two-dimensional space is 0, but the segment is not an empty set. This zero-measure implication is particularly important in the following sections, where process operability is determined by whether the achievable output set is empty. Also, if the desired input set or the desired output set is empty, a sufficiently large negative value is given to their measure to distinguish it from the nonempty set with zero measure. Thus, the measure of an empty operability set is stipulated as negative infinity. In the computational application of this operability measure, since the non-negative hypervolume condition can be checked with an if statement, this measure can also serve as an “index” for the emptiness of a set.

In the following sections, different operability sets will be defined, and the summary of the different sets that are used in this work and their corresponding notations is provided in Table 1. In particular, for the set nature “steady-state”, only steady-state values are included, and the transient dynamics are not considered. For “dynamic funnel,” the set contains all possible values from the initial time 0 until the moment immediately preceding a given time *t* or *k*. The “dynamic snapshot” denotes the set of values at a fixed time, instead of a whole time interval as in the ”dynamic funnel”.

**Table 1:** Summary of Operability Set Notations

Operability Set	Description	Nature
*AIS*	available input set	steady-state
*AIS*_*op*_	available input set for operations	steady-state
*AIS*_*des*_	available input set for design	steady-state
*AIS*^*t*^ or *AIS*^*k*^	dynamic available input set for operations from time 0 to time *t* or time *k*	dynamic funnel
*EDS*	expected disturbance set	steady-state
*EDS*^*t*^ or *EDS*^*k*^	dynamic expected disturbance set from time 0 to time *t* or time *k*	dynamic funnel
AOS	achievable output set	steady-state
*AOS*_*u*_(*d*, *x*_0_, *t*) or *AOS*_*u*_(*d*, *x*_0_, *k*)	dynamic achievable output set at time *t* or time *k* given a disturbance sequence *d* and an initial state *x*_0_	dynamic snapshot
*AOS*(*x*_0_, *t*) or *AOS*(*x*_0_, *k*)	dynamic achievable output set at time *t* or time *k* given an initial state *x*_0_ regardless of the disturbances	dynamic snapshot
*AOS*_*G*_(*x*_0_, *k*)	dynamic achievable output set at time *t* or time *k* given a feedback law *G* and an initial state *x*_0_	dynamic snapshot
*AOS*^*∞*^(*x*_0_)	time-invariant achievable output set given an initial state *x*_0_	dynamic snapshot
*DOS*	desired output set	steady-state
*DOS*(*t*)	dynamic desired output set at time *t*	dynamic snapshot
*DIS*_*des*_ or *DIS*	desired input set of all feasible designs	steady-state
*DIS*_*op*_	conventional desired input set of operational variables	steady-state

The numerical computation of the operability sets in the following sections is an adaptation of the multimodel operability framework.^9^ In this approach, space discretization techniques are employed to model the input-output mapping relationship as series of linearized models. In practice, the input sets are partitioned into a union of convex polyhedra, and the output sets are the union of the images of these input polyhedra’s projections onto the output space. Input and output polytopes in these models are connected, which simplifies the computation of space intersections and hypervolumes. By using polytopes and barycentric interpolations, the inverse model and other calculations can be performed efficiently.

### 2.1 Steady-State Operability Concepts

For a steady-state process, the time index *t* in eq 1 is removed and the time derivative *ẋ*(*t*) is set to 0. The input-output mapping for the steady-state operability analysis is simplified to the following expression:
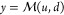
3

The objective of the steady-state operability analysis is to determine whether a desired steady-state performance requirement can be achieved considering the available inputs regardless of the realizations of the disturbances. In the following section, steady-state operability is used to find a modular unit’s feasible design region, which guarantees that the product specifications are reached in the presence of disturbances or uncertainties. Using the mapping given by eq 3, the operability sets that describe the readily accessible information and feasible results of interest are specified below.

#### Available Input Set (*AIS*)

Set of input variables that can be freely selected from a given range provided by the process constraints. In the previous operability work,^9^*AIS* was divided into *AIS*_*des*_ and *AIS*_*op*_. The *AIS*_*des*_ includes the design specifications (e.g., reactor sizes, membrane properties, etc.), and the *AIS*_*op*_ includes the operating conditions (e.g., pressure, flow rates, etc.). Mathematically, the *AIS* is defined as
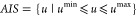
4

#### Desired Output Set (*DOS*)

Set of output variable targets that are needed for the given system. The *DOS* may contain the product specifications and the output constraints. Mathematically, the *DOS* is defined as
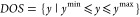
5

#### Expected Disturbance Set (*EDS*)

Set of realizations of the disturbances, which are not manipulated inputs. The *EDS* can represent uncertain parameters (e.g., activation energy, kinetic parameters, etc.) and process disturbances (e.g., ambient temperature, inconsistent feed streams, etc.). In the traditional operability definition, if only the upper and the lower bounds of the disturbances are considered, the *EDS* is given by
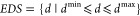
6

In this work, the disturbances, , are assumed to be in a Gaussian random vector with a mean d̅ and a covariance matrix Σ. Since a Gaussian distribution has unbounded support, the *EDS* is defined as the region of 99% highest density of the disturbances. If the disturbance is a scalar, the *EDS* is defined as the interval of six standard deviations that center at the mean. Mathematically, then the *EDS* is represented by an ellipsoid with the scale *l*^2^ equal to the inverse cumulative distribution function of the chi-squared distribution with *n*_*d*_ degrees of freedom:
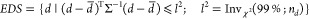
7

#### Achievable Output Set (*AOS*_*u*_(*d*))

Set of all reachable output variables given the process and the *AIS* at a fixed value of the disturbance *d* ∈ *EDS*. Mathematically, the *AOS*_*u*_(*d*) is defined as
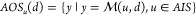
8

#### Desired Input Set (*DIS*_*u*_(*d*))

Set of all input values that map to the *DOS* when the disturbance is held at a constant value *d* ∈ *EDS*. In the traditional operability definition, *DIS*_*u*_(*d*) is defined by an inverse mapping of eq 1, which does not cover all possible inputs in the presence of input-output multiplicities. A more generalized definition of *DIS*_*u*_(*d*) is formulated here, in which its mathematical description is the following:
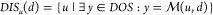
9

### 2.2 Dynamic Operability Concepts

Dynamic operability corresponds to an extension of the aforementioned steady-state operability analysis. Similar to steady-state operability, dynamic operability is the ability to reach the desired dynamic performance requirements given the input ranges and regardless of the process disturbances. While steady-state operability analysis determines the feasibility of a process design, dynamic operability gauges the effectiveness of a control structure during online operations. In other words, dynamic operability assesses whether the manipulated inputs can compensate for the effects of the disturbances during dynamic operations. In this time-varying setting, the definitions of the operability sets are extended as below.

For a dynamic process, the dynamic model given in eq 1 is simplified to the following input-output mapping:
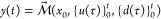
10
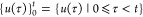
11
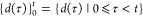
12in which *x*_0_ is the initial value of the state vector *x*(*t*), {*u*(τ)}_0_^*t*^ is the set of all control actions from the time 0 to time *t*, and {*d*(τ)}_0_^*t*^ is the set of all recorded disturbances from the time 0 to time *t*. Since the output vector, *y*(*t*), results from the initial condition, *x*_0_, the manipulated input sequence and the disturbance sequence up to time *t*, the dynamic mapping notation, , is introduced here to distinguish from the steady-state mapping notation above, .

#### Available Input Set at time *t* (*AIS*^*t*^)

Set of all inputs from the initial time 0 until the moment immediately preceding time *t* that can be freely manipulated during the operations of a given process for a given range provided by the process constraints. Dynamic operability examines the ability to change the inputs to compensate for the disturbances, but the design of a plant is not changed after its construction. For this reason, when choosing the inputs for the dynamic operability analysis, design variables (e.g., reactor sizing, materials, etc.) are not considered.
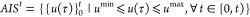
13

#### Expected Disturbance Set at Time *t* (*EDS*^*t*^):

Set of all possible values of the disturbances from the initial time 0 until the moment immediately preceding time *t*. In this framework, the process disturbances are assumed to be independent and identically distributed Gaussian random variables. Similarly to the steady-state operability *EDS*, the *EDS*^*t*^ is bounded by an ellipsoid region around the highest probability density, and its mathematical description is the following:
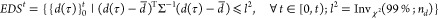
14

#### Achievable Output Set at Time *t* (*AOS*_*u*_(*d*, *x*_0_, *t*)):

Set of all reachable output vectors at time *t* from the initial condition *x*_0_ given the *AIS*^*t*^ and a fixed sequence {*d*(τ)}_0_^*t*^ ∈ *EDS*^*t*^ of the disturbances. While the dynamic operability sets in the input and disturbance spaces (*AIS*^*t*^ and *EDS*^*t*^) are defined based on a time horizon, the dynamic operability set in the output space, such as the *AOS*(*x*_0_, *t*), is defined as a ”snapshot” of a fixed moment in time. This formulation allows the *AOS*(*x*_0_, *t*) to have the same output dimensions at different times, which leads to a simpler comparison between different output sets. Mathematically, the *AOS*(*x*_0_, *t*) is defined as
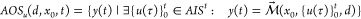
15

#### Desired Output Set at time *t* (*DOS*(*t*))

Set of output vector targets at time *t*. This set corresponds to a collection of specified control intervals for the process outputs (e.g., range of output concentrations, temperatures, etc.). The *DOS*(*t*) can be time-varying or time-invariant. A time-varying *DOS*(*t*) represents a scenario where the control objectives change with time, such as the outputs of a load-following power plant. Mathematically, a time-varying *DOS*(*t*) is a compact set that is defined by a set of time-varying inequalities:
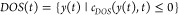
16

In this article, a time-invariant *DOS*(*t*) is assumed to be sufficient to represent the output specifications of a modular unit. Thus, the *DOS*(*t*) is simplified to a bounded set on the outputs.
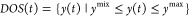
17

#### Desired Input Set at Time *t* (*DIS*_*u*_^*t*^(*d*,*x*_0_))

Set of all input sequences {*u*(τ)}_0_^*t*^ that bring the output vector from the initial state, *x*_0_, to a vector in the *DOS*(*t*) for a fixed sequence, {*d*(τ)}_0_^*t*^ ∈ *EDS*^*t*^, of the disturbance. Mathematically, the  is defined as
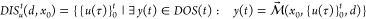
18

Due to the fact that the above operability sets are defined in continuous time, the operability mappings between the input sets, disturbance sets, and output sets may be intractable as there may exist infinitely many *AIS*^*t*^, *AOS*_*u*_(*d*, *x*_0_, *t*) in a finite time interval [0, *t*]. To circumvent this issue, the time domain of the continuous model in 1 is discretized as follows:
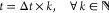
19

Since there is a diverse collection of discretization methods in the literature and the operability concepts are applicable regardless of the chosen discretization, without loss of generality, a zero-order hold is applied to the inputs and the disturbances. The input-output mapping in 10 is then obtained by approximating the solutions of the differential algebraic equations in 1. Additionally, the discrete-time operability sets are formulated similarly to their respective continuous sets above with a time index *k* instead of *t* as a superscript.

## 3 Proposed Approach

### 3.1 Proposed Framework for Design and Control of Modular Units

Modular plants consist of a series of modular units that can be independently replaced and upgraded as needed. Each modular unit is a self-contained steel structure (i.e., a module) with integrated process equipment, pipings, instrumentations, and an integrated control system.^1^ While modular units may be more efficient due to process integration and intensification, they may have fewer operational degrees of freedom.^2^ Furthermore, the manufacturing cost of modules is typically higher than for traditional units due to the additional requirement to withstand the stress of transportation and installation. To address these challenges, the upfront engineering of a modular unit has to ensure that it can be operated within a wide range of feed conditions while guaranteeing product specifications. Subsequently, mass-producing a sufficient number of modules can reduce their cost to be less than or equal to their conventional counterparts.^34^

**Figure 1:**
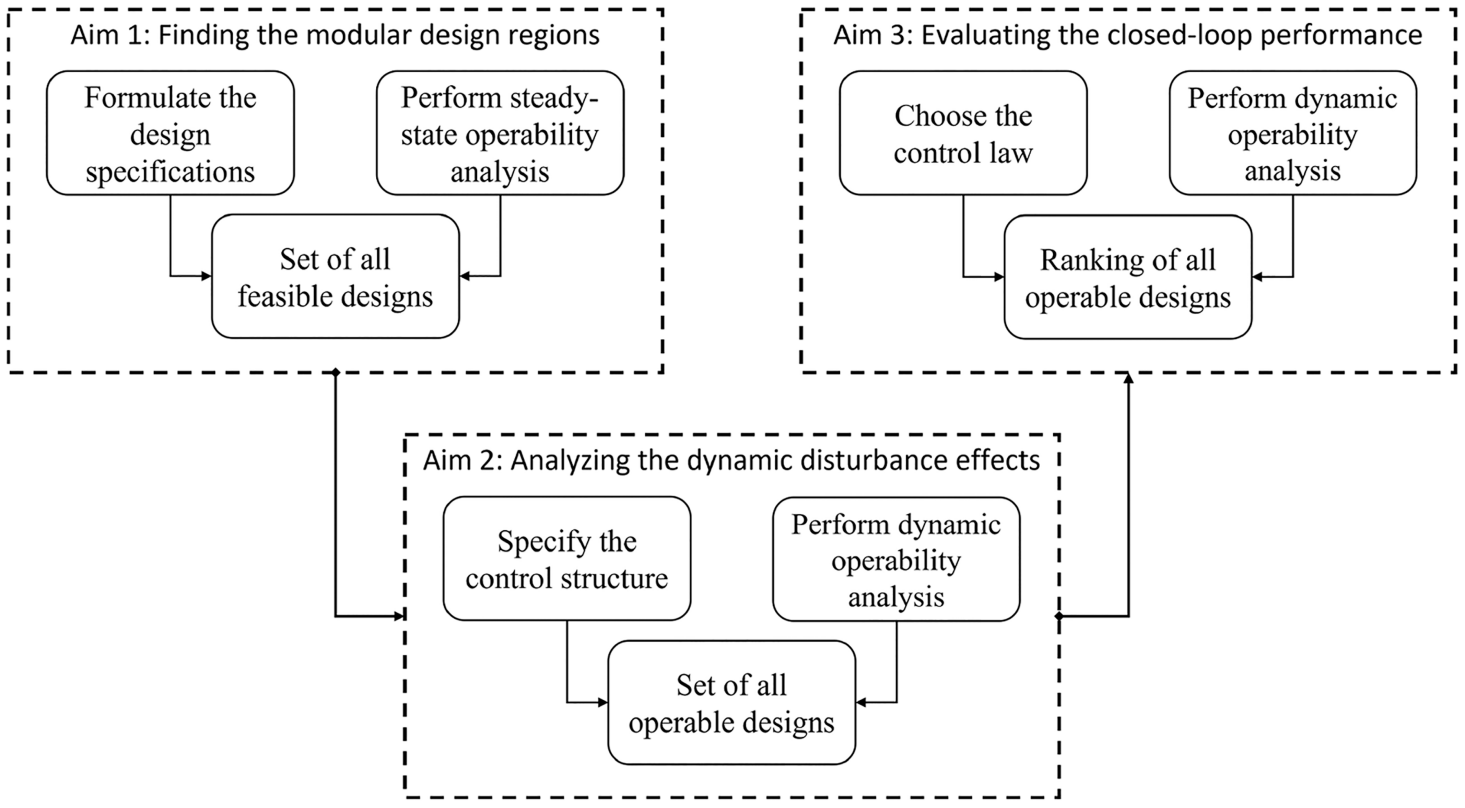
A three-step operability framework for design and control of modular units.

In this work, an operability-based framework is proposed to analyze the relationship between the design and control of a modular unit. The overall objective is to identify the set of feasible designs for a modular unit that can reach the desired product quality specifications while operating under different conditions. This objective is divided into three aims, in which each aim is discussed in the following subsections. An illustration of the proposed framework is provided in Figure 1.

The first aim is to identify all the feasible designs, for which the desired outputs are achievable considering all values of the disturbances. For process design, two sets of considered inputs are the design inputs and the operations inputs. The design inputs are the malleable inputs during the manufacturing of modular units that are not changeable during the operation. The operations inputs are the manipulated inputs during the operation, such as valve positions. Since most processes are operated around some steady-state conditions, the steady-state operability in this aim is necessary to quantify the relationships between the design and control of a modular unit. This aim is further explored in subsection 3.2.

The second aim is to find all the operable designs from the feasible designs identified in the first aim. A control structure is defined here as the selection of manipulated input variables and output variables. A control structure of a design is operable if its manipulated inputs are capable of compensating for the effects of the disturbances during the online operation. Since the operation of a modular unit corresponds to a dynamic process, the steady-state operability analysis in the previous step is not sufficient to evaluate the control structure of a process. Thus, using dynamic operability analysis for a fixed disturbance set is employed in this step. This aim is discussed in more details in subsection 3.3.

The third aim is to introduce a measure for the closed-loop performance of a modular system with a fixed control law. A control law is defined as the policy of changing the manipulated variables according to the current state of the system to achieve a predefined set point. Some examples of control laws are classical feedback PID controls, and nonlinear model predictive control (NMPC). In advanced process control such as NMPC, a layer of state estimation is usually paired with the controller to estimate the process states from the measurements. However, the replacement of the actual full state information with estimated state information does not affect the fixed control law dynamic operability analysis performed in this step. Thus, in this work, the states of a dynamic process are assumed to be available as needed, and a suggestion on the modification of this step with a state estimator is provided below. The final aim is developed in subsection 3.4.

### 3.2 Steady-State Operability Analysis for Feasible Design Region

A feasible design is considered to be a design of a modular unit that has some steady-state outputs contained within the *DOS* given by eq 5 for all values of the process disturbances in the *EDS*. The ranges of the design variables in the *AIS* are given by the manufacturing capability of the modular unit’s production facility and the shipment requirements of the transportation method, such as container trucks or freight trains. The *DOS* is assumed to be a set of output specifications that the targeted modular plant will have to meet. Since identical modular units are potentially shipped to build modular plants that will be operating at different conditions, the *EDS* represents different manufacturing conditions and external disturbances that can be defined and characterized before the construction of the modular plant. Once the *AIS*, *EDS*, *DOS*, and the steady-state mapping are defined, the *AOS*_*u*_(*d*) and *DIS*_*u*_(*d*) sets can be obtained for every fixed disturbance value *d*.

In the previous multilayer operability framework,^9^ the steady-state operability analyses are sequentially performed on the *AIS*_*des*_ and the *AIS*_*op*_ to find a feasible design region and to rank different designs based on the operations. However, by excluding the operational variables from the *AIS*_*des*_, only the nominal outputs instead of not all achievable outputs are covered in the design’s operability analysis. In this work, the steady-state operability analysis is generalized to include the *AIS*_*des*_ and optionally the *AIS*_*op*_. In the steady-state forward mapping from the input space to the output space, an output vector represents a feasible steady-state condition. Since the considered modular process given by eq 1 is dynamic, a feasible steady-state condition exists, but the process may never be able to be constantly maintained at that steady-state condition during transient due to the presence of disturbances. However, if a steady-state condition does not exist (or is unreachable from the given *AIS*), the outputs of the dynamic process are guaranteed to never approach the neighborhood of the infeasible (or nonexisting) steady-state condition, and the following dynamic operability analyses are not needed. Thus, the purpose of the steady-state operability analysis is not to find the set of all feasible designs, but to eliminate all infeasible designs under the most ideal conditions of disturbances and operational inputs. For this reason, design variables must be included in the steady-state *AIS*, but the inclusion of operational variables in the *AIS* is optional.

Since a design is immutable after the manufacturing of a modular unit is finished, the choice of a steady-state achievable output can be freely selected from the *AOS*_*u*_(*d*), but it is fixed permanently after being chosen. If operational inputs are included in the steady-state *AIS*, an achievable output is considered as a steady-state set point, which is also fixed with respect to the established design. Additionally, the *AIS* and the *AOS*_*u*_(*d*) only represent the boundaries of the achievable designs and the achievable steady-state outputs without revealing exactly which design maps to which output. As a result, comparing all different *AOS*_*u*_(*d*) against the *DOS* reveals no information on which design is feasible regardless of the disturbance realization in the *EDS*. However, the inverse mapping of the *DOS* to different *DIS*_*u*_(*d*) in the input space distinguishably identifies the achievable desired input set (*DIS*_*des*_) via the following set intersection:
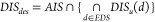
20

In the traditional operability concept,^20^ only the *AIS*_*op*_ is considered for the steady-state operability analysis. Unlike the input in the *AIS*_*des*_, inputs in the *AIS*_*op*_ are changeable after a modular unit is manufactured. Thus, the traditional definition of the *DIS*, or *DIS*_*op*_ in this work, is an extended input region of the manipulated variables that is needed to reach all the points in the *DOS*. Mathematically, the traditional *DIS*_*op*_ is formulated as following:
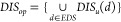
21

In the current article, the *DIS* notation is simplified to only denote the *DIS*_*des*_ in eq 21. As the *DIS* is the subset of the *AIS* that always maps to the outputs in the *DOS* regardless of the value of *d* ∈ *EDS*, the *DIS* by definition is the set of feasible designs for all values of the disturbance. If the feasible design region does not exist, then the *DIS* is an empty set, and one of the following scenarios can be considered. If the intersection of all *DIS*_*u*_(*d*) is nonempty, then the range of the design variables should be increased such that the *AIS* has some overlap with this intersection. If the intersection of all *DIS*_*u*_(*d*) is empty, an alternative modular design should be considered. Mathematically, the feasible design region exists if its μ measure from eq 2 is non-negative:
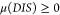
22

**Figure 2:**
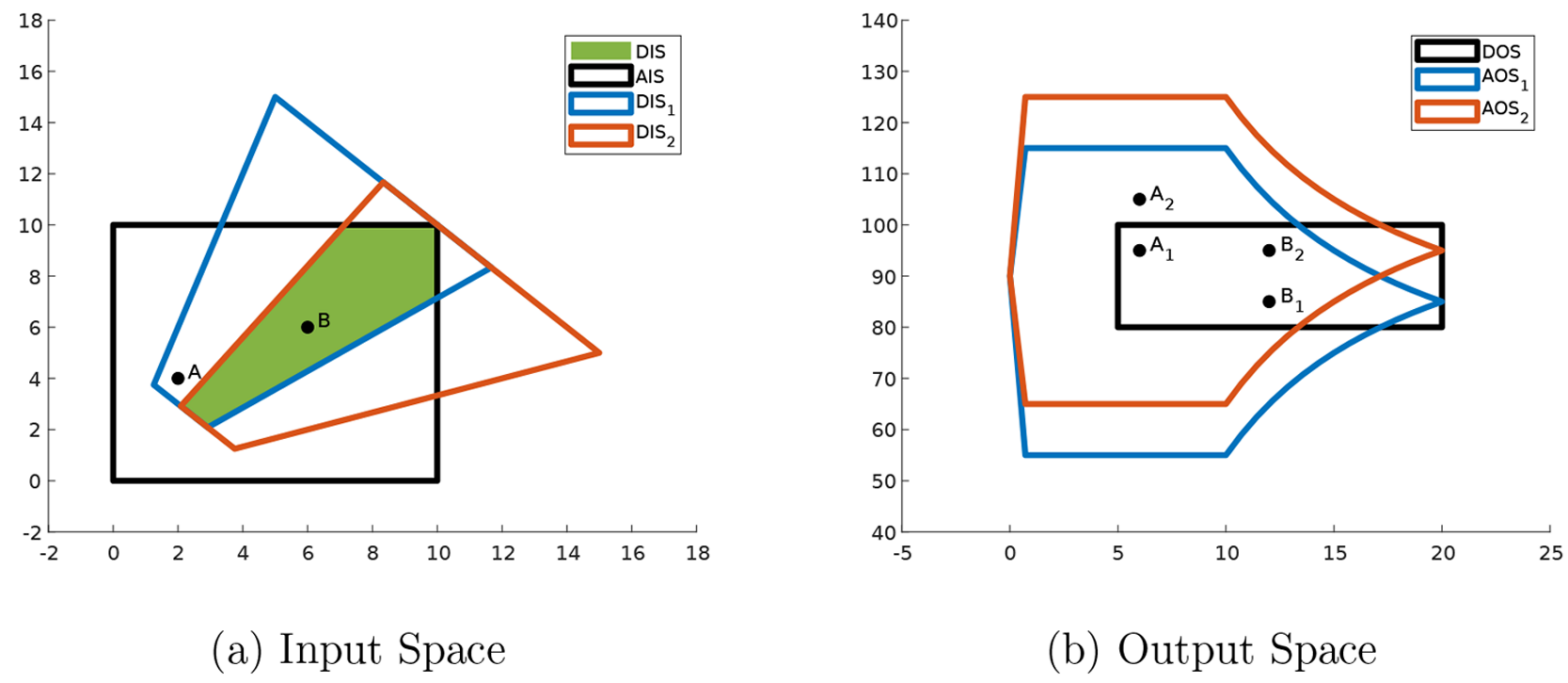
Steady-state operability analysis schematic to find the feasible design region.

An example of steady-state operability analysis to find a feasible design region is illustrated in Figure 2. The *AIS* in the example represents two design variables that cannot be changed after being chosen. Two different designs, *A* and *B*, are selected, and each design is represented by their respective point in Figure 2a. The *EDS* in this illustrative example is assumed to contain two values, *EDS* = {1,2}, and the achievable outputs of each design are indexed by a subscript of the disturbance value. In Figure 2b, *A*_1_ and *A*_2_ are respectively the achievable outputs of the design *A* when the disturbance takes the value of 1 and 2. The achievable outputs *B*_1_ and *B*_2_ are defined similarly. Since design *B* is contained in the *DIS*, both of its achievable outputs lie in the *DOS* regardless of the value of the disturbance, and design *B* is thus a feasible design. On the contrary, the disturbance shifts the achievable output of design *A* outside the *DOS*, so design *A* is not a feasible design. Let the images of the *AIS* at disturbance values of 1 and 2, respectively, be *AOS*_1_ and *AOS*_2_; the intersection of *AOS*_1_, *AOS*_2_, and *DOS* is not sufficient to identify the set of all feasible designs. Without an inverse mapping, the achievable outputs *A*_1_ and *B*_1_ both belong to the aforementioned intersection, but only design *B* is feasible. Thus, the set of all feasible designs are the intersection of the *AIS* and *DIS*_1_ and *DIS*_2_, which are respectively the inversely mapped image of the *DOS* at disturbance values 1 and 2.

### 3.3 Dynamic Operability Analysis for Operable Region

The dynamic operability concept is an extension of the controllability concept in modern control theory. A process is controllable if there always exists a manipulated input sequence to reach any arbitrary point in the output space in finite time.^35^ While controllability is well-defined with consistent mathematical conditions, such as the full-rank condition of a controllability matrix for a linear system, this property is not always applicable to an actual control system with input constraints, output constraints, and process disturbances. To bridge this gap between controllability and the operations of physical systems, dynamic operability is defined as the ability to reach a *DOS*(*k*) in a finite time given a manipulated input sequence from *AIS*^*k*^ considering the effects of the disturbances. In other words, a dynamic process is fully operable if there is a manipulated input sequence that satisfies the constraints and brings the outputs to desirable values for all scenarios of the disturbances. Similar to controllability, dynamic operability is an inherent property of the system, and it is independent of the formulation of the selected control laws.

The achievable output set (*AOS*(*x*_0_, *k*)) at a discrete-time *k* from an initial condition *x*_0_ of the dynamic process given by eq 1 is mathematically defined as
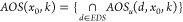
23

Unlike the design inputs for the steady-state *AIS*, the manipulated inputs can be moved in the *AIS*^*k*^ at any given time. Each *AOS*_*u*_(*d*, *x*_0_, *k*) at a fixed *d* represents all possible values of the outputs by manipulating the inputs, and the transformations of *AOS*_*u*_(*d*, *x*_0_, *k*) according to *d* correspond to the effect of the disturbances on the outputs. Thus, every output in the *AOS*(*x*_0_, *k*) is achievable if the exact disturbance sequence {*d*(τ)}_0_^*k*^ is provided at time 0. In practice, {*d*(τ)}_0_^*k*^ is only available at time *k*, so the *AOS*(*x*_0_, *k*) represents the best attempt of a controller to reject the predicted disturbances. Furthermore, a process is dynamically operable if its *AOS*(*x*_0_, *k*) always overlaps with the *DOS*(*k*) after a finite time value *k̂*. Mathematically, the dynamic operability condition is thus defined as follows:
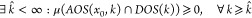
24

**Figure 3:**
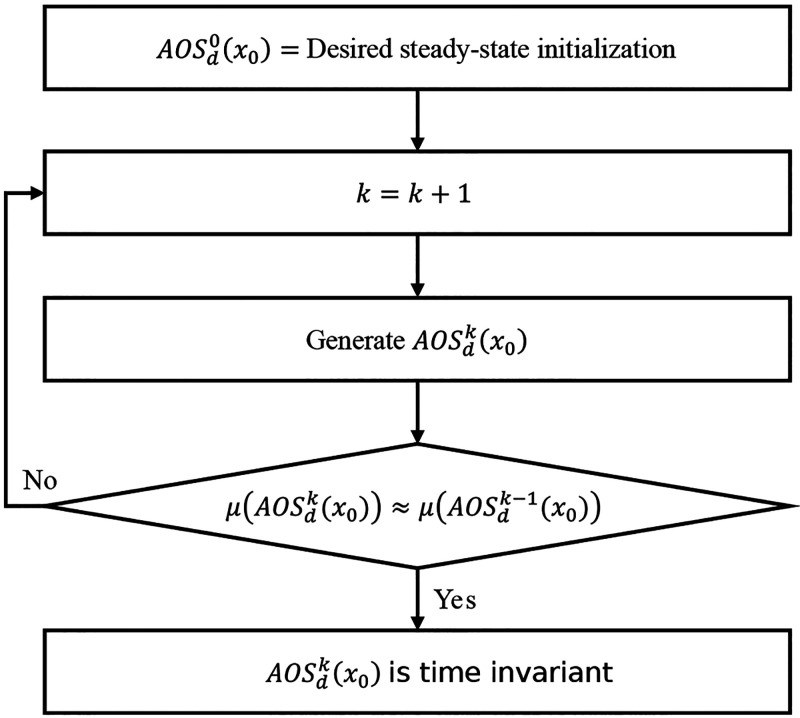
Procedure of finding the time-invariant *AOS*^*∞*^(*x*_0_).

According to the condition in eq 24, dynamic operability analysis can be computationally intractable since the *AOS*(*x*_0_, *k*) at all values of *k* are needed. Fortunately, if the process given in eq 1 is input-to-state stable,^36^ the *AOS*_*u*_(*x*_0_, *k*) converges to a time-invariant set *AOS*^*∞*^(*x*_0_) as *k* approaches *∞*. Since the disturbances can be considered as random inputs of a process, the disturbances can be defined as admissible inputs for the stability analysis. Therefore, for any fixed value of the disturbance and manipulated inputs, the outputs are covered by a compact set. Thus the set intersections in eqs 23 and 24 are also bounded by compact sets. Additionally, the generation of *AOS*_*u*_(*d*, *x*_0_, *k* + 1) in eq 23 is equivalent to the union of all one-step mappings with an initial condition drawn from *AOS*_*u*_(*d*, *x*_0_, *k*). As a result, the measure μ(*AOS*(*x*_0_, *k*)) is always less than or equal to the measure μ(*AOS*_*u*_(*d*, *x*_0_, *k* + 1)). Because the measure μ(*AOS*_*u*_(*d*, *x*_0_, *k*)) is increasing with *k* and bounded above, it converges to a constant value. Mathematically, the time-invariant set *AOS*^*∞*^(*x*_0_) is reached at time *k*^max^ if the difference in measure of two consecutive *AOS*_*u*_(*d*, *x*_0_, *k*) is less than a predefined threshold ε:
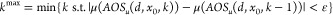
25
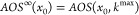
26The time-invariant set *AOS*^*∞*^(*x*_0_) can be found by generating μ(*AOS*(*x*_0_, *k*)) one at a time until the threshold ε on the difference in measure μ is met. Since the μ(*AOS*(*x*_0_, *k*)) will always converge to the same *AOS*^*∞*^(*x*_0_) regardless of different initializations *x*_0_, a steady-state solution from the feasible region obtained in eq 20 is chosen. An illustration of this procedure is provided in Figure 3.

The dynamic operability concept in this subsection can be extended to obtain a concept of stochastic operability. If the *EDS*^*k*^ given by eq 14 is limited to the highest density region of probability, α, instead of 99%, the *AOS*^*∞*^(*x*_0_) will represent the set of outputs that can be achieved for at least α percent of the disturbance values.

Note that in this, the dynamic operability analysis is dependent on the initial state *x*_0_, and the role of the initial state falls into one of the following two scenarios. In the first scenario, the initial state can be manipulated by the operators, and the *AIS*^*t*^ is expanded to include *x*_0_ as a manipulated input. An example of this scenario is the start-up of a modular process. In the second scenario, the initial state is a random vector with bounded support, and the *EDS*^*t*^ is expanded to include *x*_0_ as an additional process disturbance. An example of this scenario is when dynamic operability mapping is performed along with the online operation, and the states are deviated by the process disturbances. In both scenarios, the dynamic operability can be performed as presented above with the modification of the *AIS*^*t*^ or the *EDS*^*t*^.

### 3.4 Closed-Loop Performance Measure with Dynamic Operability

The result of the steady-state operability analysis is a feasible design region. From each point of the feasible design region, dynamic operability analysis is performed to find the operable design region that is a subset of the feasible design region. While this is sufficient for a modular design to meet output specifications at different modular plant conditions, an optional step for measuring the closed-loop control performance for a fixed control law is provided in this subsection. The closed-loop analysis performed here is a special case of the dynamic operability analysis proposed in subsection 3.3.

Consider the following control law:
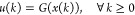
27in which *G* is a feedback control law. Since the manipulated inputs are determined by the states, they are dependent on the state measurements and are no longer freely available to be selected, and thus the set *AIS*^*t*^ is not considered. *The Achievable Output Set at time k with fixed control law G* (*AOS*_*G*_(*x*_0_, *k*)) is the set of all possible outputs as a result of the disturbances given in *EDS*^*k*^.
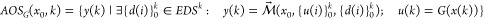
28

When the process is input-to-state stable, the time-invariant  is obtained with the procedure in Figure 3, similarly to *AOS*^*∞*^(*x*_0_). Since *AOS*_*G*_(*x*_0_, *k*) corresponds to the set of output deviations caused by the process disturbance, the measure  represents the hypervolume of the fluctuations of the process under closed-loop control.

The summary of the differences between dynamic mappings and operability analyses in subsection 3.3 and subsection 3.4 is provided in Table 2.

**Table 2:** Comparison between Different Dynamic Operability Mappings

	Aim 2: Dynamic mapping with fixed disturbance	Aim 3: Dynamic mapping with fixed control law
Objective	The objective is to analyze a control structure of a dynamic system by assessing whether the process disturbances are sufficiently rejected	The objective is to analyze the closed-loop behaviors of a fixed controller by measuring the fluctuations in the process outputs
Characteristics	Dynamic mapping is independent of the formulation of the controller	Dynamic mapping is defined by a fixed controller formulation
Available Input Set	The input set is the range of the manipulated variables	The manipulated control inputs are dependent on the state of the process, so the input set is not considered
Achievable Output Set	The output set contains all reachable outputs via manipulating the control variables	The output set contains all possible displacements of the outputs due to the process disturbances
Expected Disturbance Set	The disturbance set is the range of process disturbances	The disturbance set is the range of process disturbances

If a layer of state estimation is considered in combination with the fixed control law *G*, the estimation errors can be a source of fluctuations in the closed-loop performance. Thus, the estimation error can be added to the *EDS*^*k*^, and the above closed-loop analysis is executed as proposed in this subsection.

## 4 Modular Membrane Reactor Case Study

**Figure 4:**
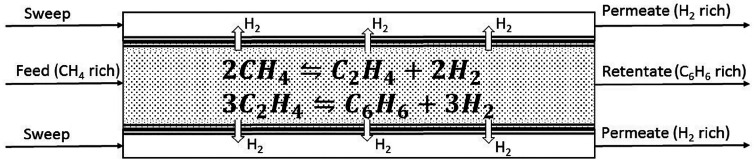
Schematic with inputs, outputs, and reactions of the DMA-MR.

The proposed framework is demonstrated via a case study of a DMA-MR, which is an intensified reaction-separation unit that converts methane in natural gas to hydrogen and benzene. The reactions are carried out in the tube of the shell-and-tube DMA-MR design, and the membrane is highly selective toward hydrogen permeation. When hydrogen is removed from the reactive tube, the reaction equilibrium shifts toward the products resulting in higher methane conversion. A schematic of a cocurrent flow configuration of the DMA-MR is provided in Figure 4.

For this study, a modular DMA-MR is assumed to be part of a modular natural gas utilization plant in the Marcellus Shale region. Since the natural gas extracted in this region corresponds to the largest recoverable shale gas reservoir in the United States,^37^ a feasible modular gas processing design for this region would be a promising candidate for mass production. The following assumptions are considered for the operability analysis of the modular DMA-MR:

- A modular DMA-MR is assumed to be transported by commercial trucks, so its design is limited by the dimensions of its shipping container;^9^
- At different natural gas feeds, an identical modular DMA-MR is capable of producing sufficiently high benzene and hydrogen concentrations in the product streams, so that it can be fit for large-scale manufacturing;
- During online operation, uncertainties in the natural gas concentrations caused by upstream processes should not affect the achievability of the desired output specifications of the DMA-MR.

### 4.1 Dynamic Modeling of the DMA-MR

The dynamic model of the DMA-MR is considered to be temperature and pressure controlled, i.e., the DMA-MR model is assumed to be isothermal and isobaric. The pressure drops are neglected, and the flow rate is driven by the pressure profiles in the tube and the shell. Radial and angular symmetries are adopted, and only the change of states along the length of the DMA-MR is considered. Additionally, the equation of states is assumed to be the ideal-gas law.^38^ The dynamic model of the DMA-MR is a system of partial differential equations with differential independent variables as the time and length of the DMA-MR. To solve this model, the method of lines is applied to discretize the length of the DMA-MR into equal partitions of differential length Δ*z*. The resulting dynamic model of the DMA-MR is a system of ordinary differential equations with respect to time.

In particular, for the nonoxidative conversion of methane, the following two-step reaction mechanism is adapted from the published literature:^7,28,39^Step 1:
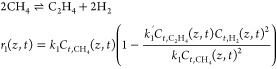
29Step 2:
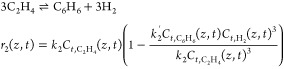
30in which *z* indexes the spatial locations along the discretized length of the DMA-MR and *t* indexes the process time of all variables, *r*_1_ and *r*_2_ are respectively the reaction rates of steps 1 and 2, *k*_1_ and *k*_2_ are respectively the forward reaction constants of steps 1 and 2, *k*_1_^′^ and *k*_2_^′^ are respectively the inverse reaction constants of steps 1 and 2, and *C*_*i*_ are the concentrations of the species *i* in the tube.

The rate of reactions of each species is calculated based on the stoichiometry of steps 1 and 2 as shown below.
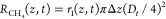
31
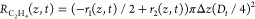
32
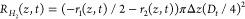
33
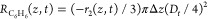
34in which *R*_*i*_ are the reaction rates of species *i* in the tube, and *D*_*t*_ is the tube diameter.

The permeations of each species through the membrane are driven by the partial pressure gradients between the tube and the shell. For an ion-based perovskite membrane, the membrane flux expression is proportional to the difference of the partial pressure gradient raised to the power of 1/4.^40^ The mass fluxes are represented by
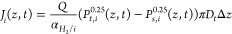
35in which *J*_*i*_ is the molar flux of species *i* from the tube to the shell, *P*_*t*,*i*_ is the partial pressure of species *i* in the tube, *P*_*s*,*i*_ is the partial pressure of species *i* in the shell, *D*_*t*_ is the tube diameter, *Q* is the H_2_ permeance through the membrane, and  is the membrane’s selectivity between H_2_ and component *i*.

At the inlets of the DMA-MR, the total molar flow rate of the tube is denoted as *F*_*t*,0_, and the total molar flow rate of the shell is denoted as *F*_*s*,0_. Using the ideal-gas equation of states, the total molar concentrations in the tube *C*_*t*_ and shell *C*_*s*_ are calculated according to the reactor temperature *T*, the tube pressure *P*_*t*_, and the shell pressure *P*_*s*_. From the isobaric and isothermal assumptions and disregarding process start-up, the total concentrations are constant with respect to time and reactor length. The following molar constraints are always active in the dynamic model:
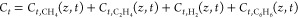
36
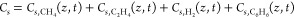
37From the ideal-gas law assumption, the molar flow profiles in the tube and the shell of the DMA-MR are scaled linearly with the molar concentrations, as shown below.
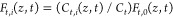
38
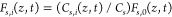
39
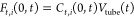
40
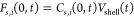
41in which *F*_*t*,*i*_ and *F*_*s*,*i*_ are, respectively, the molar flow rate of species *i* in the tube and in the shell, *V*_tube_ and *V*_shell_ are, respectively, the volumetric flow rate of species *i* at the inlet of the tube and the inlet of the shell.

Thus, the mass balances of the dynamic model for the DMA-MR are given by the following ordinary differential equations for each species:
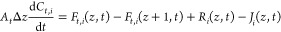
42
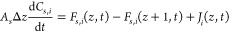
43in which *A*_*t*_ and *A*_*s*_ are, respectively, the cross-sectional areas of the tube and the shell. The parameters of the dynamic model are adapted here from the existing steady-state model in the literature.^41^

The dynamic model of the DMA-MR in this subsection is formulated for equation-oriented platforms, such as MATLAB and Python. Specifically, the time evolution of the model is solved with the *odeint* subroutine in Python or the *ode15s* subroutine in MATLAB.

### 4.2 Feasible Design Region of the DMA-MR

Starting from the dynamic model of the DMA-MR, the steady-state model was constructed by setting the left-hand sides of eqs 42 and 43 to zero. The steady-state model is thus a system of nonlinear equations, that is solved using the MATLAB’s subroutine *fsolve*. In this subsection, a feasible design of the DMA-MR is obtained from the steady-state operability analysis proposed in subsection 3.2. The objective of the analysis in this subsection is to find the set of DMA-MR designs that guarantee sufficiently high product concentrations when operating around different natural gas wells at the Marcellus Shale.

The steady-state *AIS* in this case has two design variables and one optional manipulated variable as operability inputs. The two design variables are the tube diameter and the length of the DMA-MR. Since the tube of the DMA-MR is inserted in the shell, the shell diameter is chosen to be 10 cm larger than the tube diameter in all simulations. The considered disturbance corresponds to the methane concentrations at different natural gas wells, and the disturbance range is defined based on historical data.^37^ The design specifications for the steady-state analysis are listed in Table 3.

**Table 3:** Steady-State Operability Sets of the DMA-MR: *AIS*, *DOS*, and *EDS*

Input Variables	Available Ranges
Length (*dm*)	50–100
Diameter (*dm*)	1–5
Feed flow rate of tube (*dm*^3^/*h*)	500–1000

Output Variables	Desired Ranges
benzene mole percent in tube outlet (%)	>5
hydrogen mole percent in shell outlet (%)	>17

Disturbance	Expected Range
Feed methane mole percent (%)	78–93

**Figure 5:**
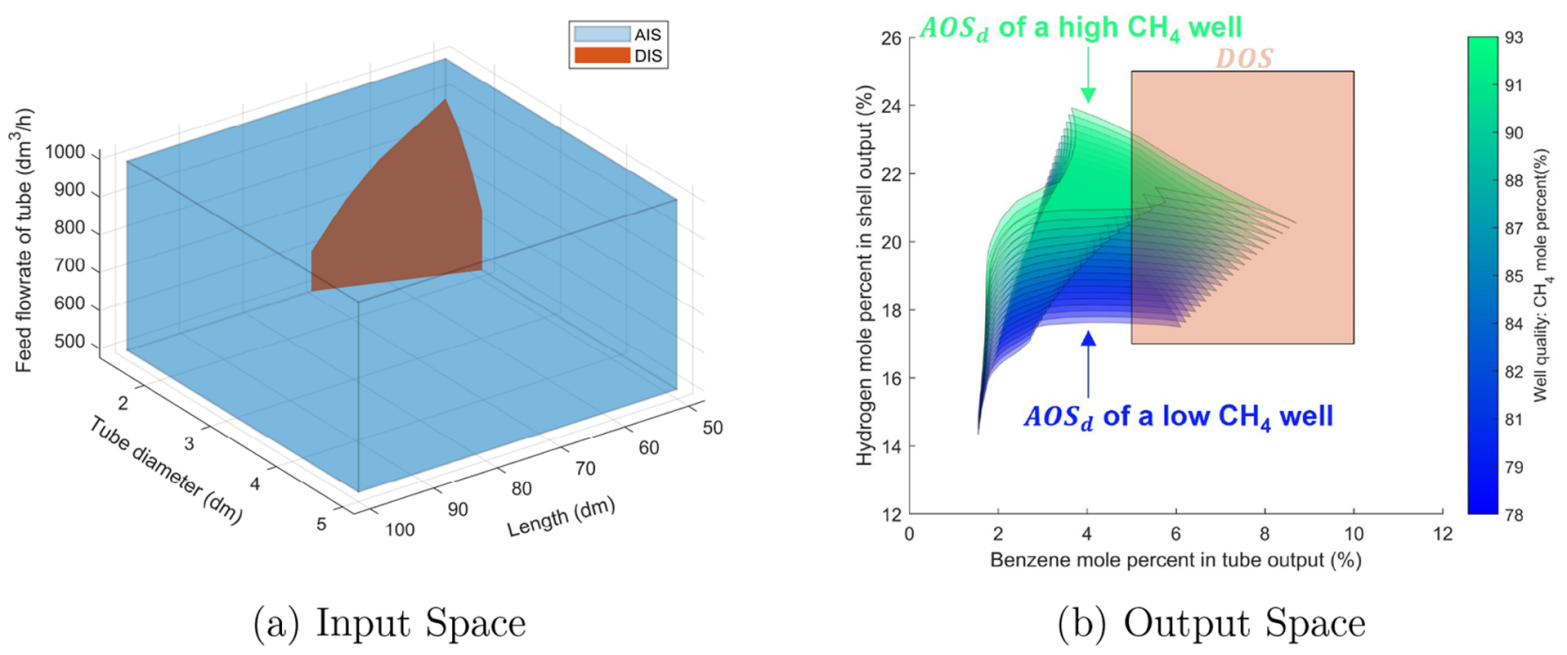
Steady-state operability analysis to find the feasible design region of the DMA-MR.

For the performed analysis, each variable range of the *AIS* and the *EDS* is partitioned into 20 equal segments. Each *AOS*_*u*_(*d*) is obtained by solving the steady-state model considering all combinations of the designs in the *AIS* at every value of the disturbance in the *EDS*. The blue polytope in Figure 5a is the *AIS* considered for the steady-state operability analysis. In Figure 5b, the results of the forward input–output mapping are 20 sets of all reachable product concentrations of the DMA-MR for different natural gas wells. In this case study, the outputs of the *DOS* are molar concentrations, so it is implied that their upper bounds cannot exceed 100%. For illustrative purposes, Figure 5b does not show the whole DOS, because the *AOS*_*d*_ would be disproportionally smaller. For each value, *d*, of the disturbance in natural gas concentration, *DIS*_*u*_(*d*) is the *AIS* subset that contains all designs of the DMA-MR that can reach the desired output concentration, which is represented by the *DOS*. The intersection of all *DIS*_*u*_(*d*) results in the feasible design region *DIS*, as illustrated in Figure 5a.

**Figure 6:**
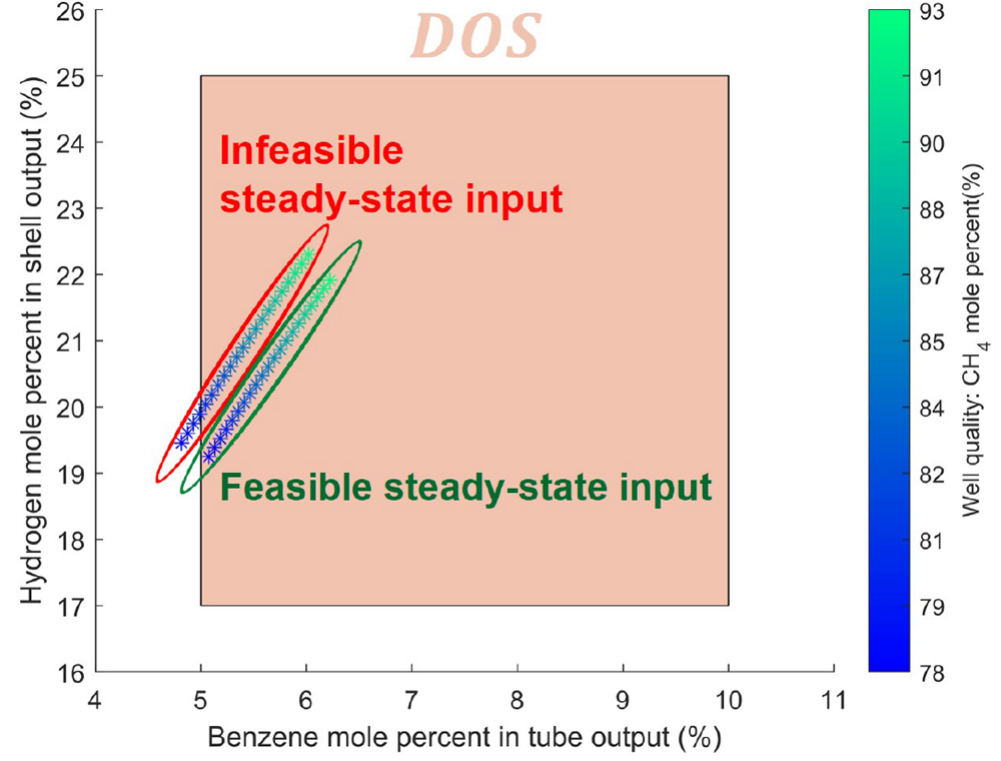
Feasibility of fixed DMA-MR designs at different natural gas wells.

In Figure 6, the significance of the feasible design region is demonstrated. This figure compares the impacts of the disturbance on an infeasible steady-state design and a possibly feasible steady-state design. For each design, different disturbance values shift the steady-state outputs along a segment, and a feasible design is the one with the segment contained within the *DOS*. Specifically, if an infeasible steady-state design is chosen for the DMA-MR, that is a point in the *AIS* but not in the *DIS*, then the benzene percentage is not sufficiently high to be included in the *DOS*, for example, the case in which this membrane reactor is operated at a natural gas well with low methane concentrations. Because the length and diameter of the DMA-MR will not change after leaving the manufacturing facility, the feasible design region is defined as the set of DMA-MR with output specifications that remained inside the *DOS* considering the *EDS*.

### 4.3 Operable Design Region of the DMA-MR

The feasible design region of the DMA-MR given by the *DIS* only reflected the ability of the system to operate at a steady-state condition, so dynamic operability is needed to evaluate whether the modular membrane reactor is now capable of rejecting the disturbance after installation at a modular gas processing plant. For the dynamic operability analysis, the manipulated variables of the DMA-MR are the inlet volumetric flow rates of the tube and the shell. The range of the inlet tube flow rate in the *AIS*^*k*^ obtained considers 20% deviations of the steady-state values from the steady-state *DIS*, and the range of the inlet shell flow rate in the *AIS*^*k*^ is chosen between 1000 dm^3^/h and 1400 dm^3^/h. The time-varying disturbance of the *EDS*^*t*^ corresponds to a normally distributed random methane concentration in the feed stream with 82% mean and 2.5% standard deviation, which is estimated from the characterization of the natural gas wells in the Marcellus region.

**Figure 7:**
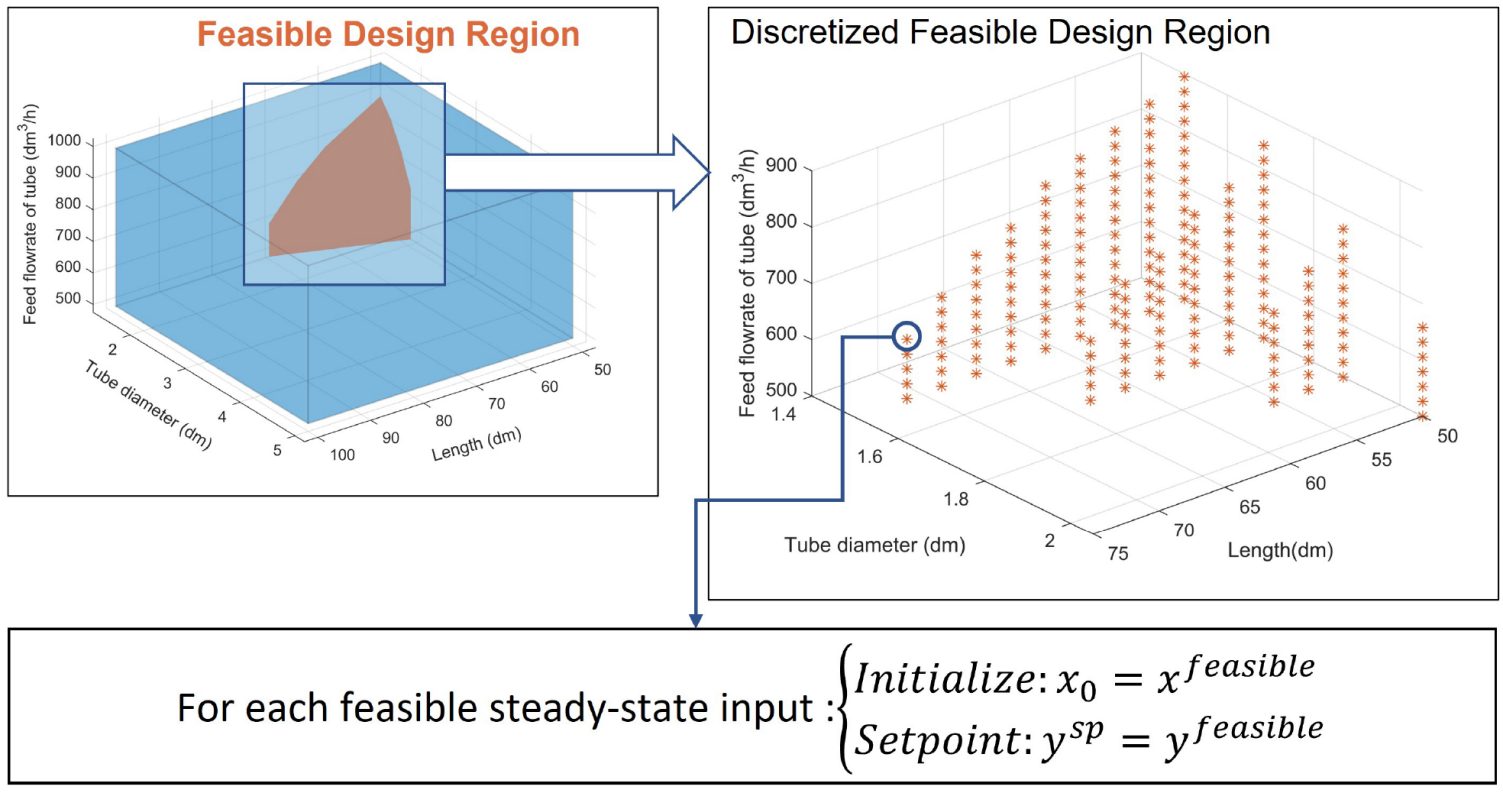
Initialization of dynamic operability analysis from the results of the steady-state operability analysis.

All simulations of the dynamic operability analysis are initialized with their respective steady-state inputs from the *DIS*. In the following subsection 4.4, the start-up and shut-down of the modular unit are not considered, and the controller objective is simply disturbance rejection operation once the membrane reactor reaches the desired steady-state. Therefore, the feasible steady-state solutions from the steady-state operability analysis of the DMA-MR are used as both the initial conditions and the set points for the MPC in the dynamic operability study. To simplify the notation, the dependency on *x*_0_ is implied from here on and thus removed from the dynamic operability sets of the DMA-MR. The initializations of the dynamic operability analyses are shown in Figure 7.

**Figure 8:**
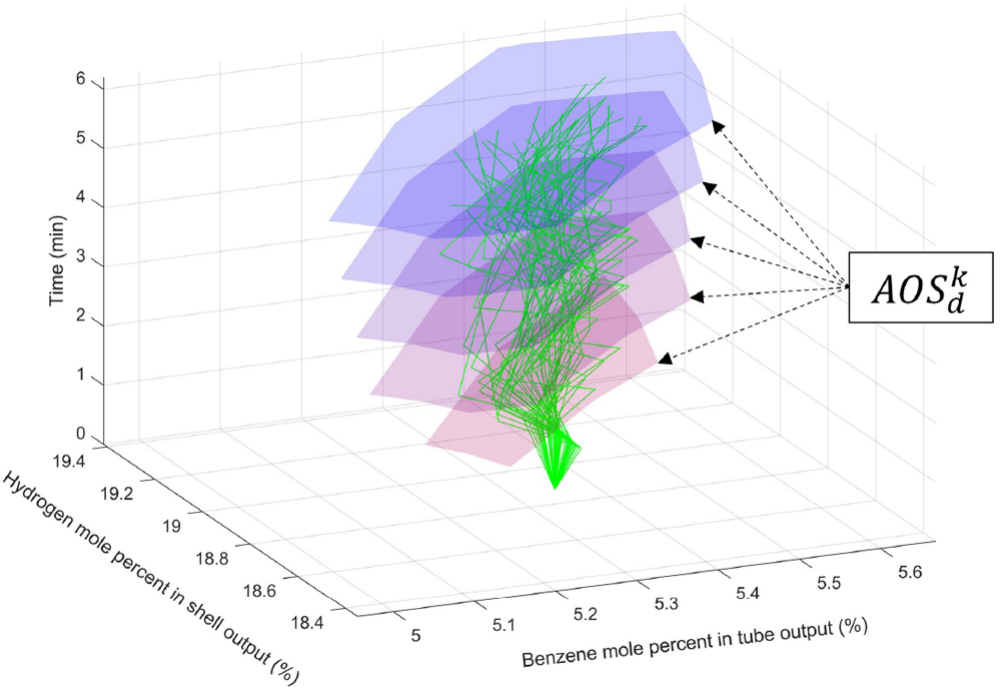
Dynamic achievable output sets for a fixed value of the disturbance.

For every time step, the inlet flow rates and the inlet methane concentration could take any value within their given ranges. For the dynamic operability mapping, each manipulated input is discretized into 5 evenly spaced points between their boundaries, and each time-step requires (5 × 5) simulations for two manipulated inputs. The threshold ε for the time-invariant *AOS*^*∞*^ calculated using eq 25 is set to 0.1, and the discrete-time difference Δ*t* in eq 19 is chosen to be 1 minute. As a result, all *AOS*_*u*_(*d*, *k*) reached their time-invariant sets in 8 discrete-time steps, and each set of *AOS*_*u*_(*d*, *k*) required (5 × 5)^8^ ≈ 4.29 × 10^6^ simulations. This is due to the fact that the number of simulations increases as a scenario-tree for the inputs as the time grows. Because the number of simulations increases exponentially with the number of time steps, the 8-step discrete-time horizon to reach time-invariancy is found using a trial and error method for one fixed design, and it is then applied to all dynamic operability mapping cases of feasible designs. Figure 8 shows a set of *AOS*_*u*_(*d*, *k*) at the mean value of the disturbance sequence, in which the reactor length is 70 dm, the reactor diameter is 4 dm, and the time-zero steady-state has the nominal natural gas flow rate of 500 dm^3^/h. The red and purple color gradients of the AOS index the time differences, and the green lines are the Monte Carlo simulations of randomly selected available manipulated inputs to demonstrate that the time-varying *AOS*_*u*_(*d*, *k*) covers all possible values of the outputs.

**Figure 9:**
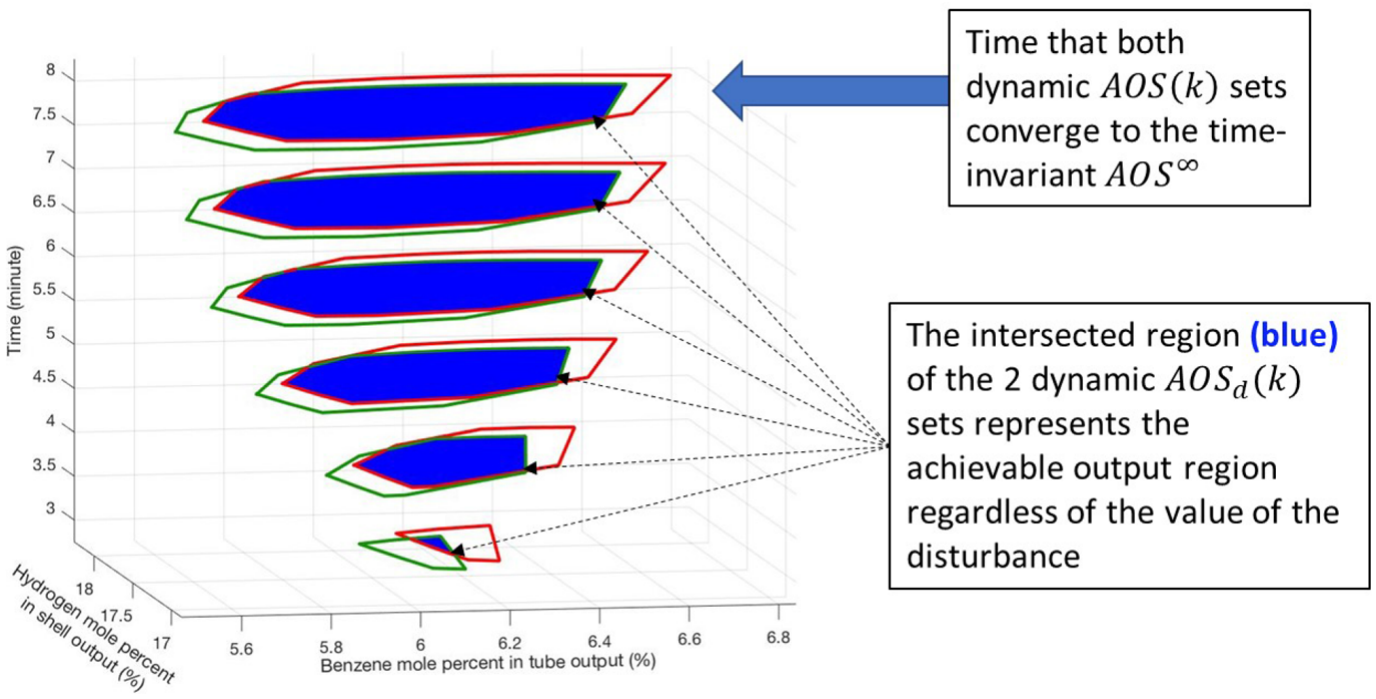
Dynamic operability analysis for two disturbance sequences.

Before the full disturbance range in the *EDS*^*k*^ is considered, a simplified case of dynamic operability is provided in Figure 9 to demonstrate the analysis proposed in subsection 3.3. In this example, the *EDS*^*k*^ only has two sequences {*d*(*i*)}_0_^*k*^ of the disturbance. The first disturbance sequence results in the set of *AOS*_*u*_(*d*, *k*) that is illustrated by the green bordered polytope funnel, and the second disturbance sequence results in the set of *AOS*_*u*_(*d*, *k*) that is illustrated by the red bordered polytope funnel. Each funnel is generated until both sets of *AOS*_*u*_(*d*, *k*) reach their time-invariant set. For every output *y*(*k*) in the intersection of both *AOS*_*u*_(*d*, *k*) sets, which are illustrated with a blue-filled polytope funnel, there exists a sequence of input manipulated variables {*u*(*i*)}_0_^*k*^ for each disturbance sequence that map to *y*(*k*). Thus, the blue funnel is the set of all achievable outputs regardless of the assumed disturbance sequences.

**Figure 10:**
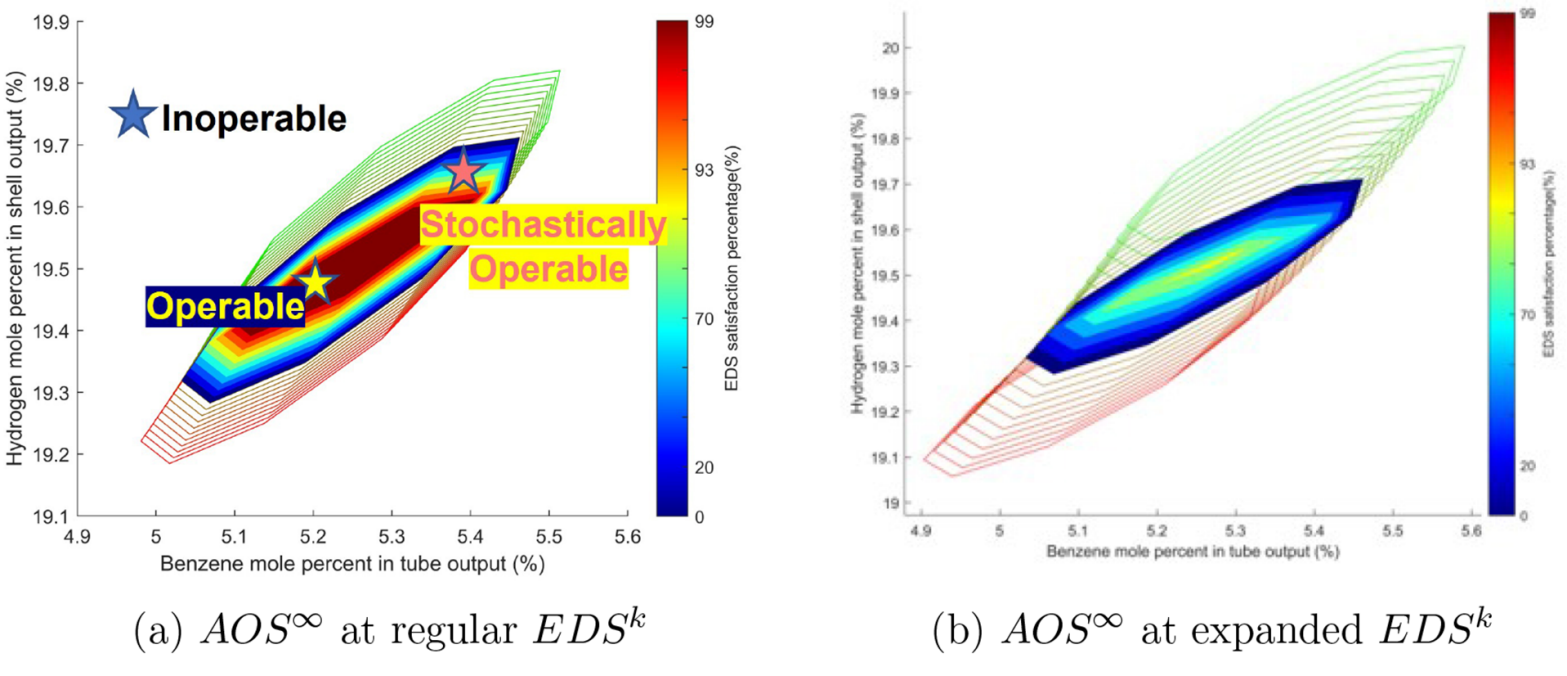
Effects of *EDS*^*k*^ on the dynamic operability via the intersections of *AOS*_*u*_^∞^(*d*).

For the complete dynamic operability analysis, the *EDS*^*k*^ is discretized at each time step *k* by partitioning the 99% highest density interval of the disturbance into 5 evenly spaced values. Since the disturbance at every time *k* is assumed independent and identically distributed, the *EDS*^*k*^ included 5^8^ ≈ 3.9 × 10^5^ disturbance sequences. At every time step *k*, the intersection of all *AOS*_*u*_(*d*, *k*) at different disturbance sequences is done similarly to the simplified case. In Figure 10, *AOS*_*u*_^*∞*^(*d*) are represented by the empty-filled polytopes with the boundary’s color gradients indicating different values of the disturbance sequences. The resulting time-invariant *AOS*^*∞*^ sets are shown as filled polytopes, and their colors represent the ranges of a fixed-time disturbance. A design of a modular DMA-MR is operable if the intersection, which is illustrated as the red-filled polytope, of all *AOS*_*u*_^*∞*^(*d*) has a nonnegative μ measure. If an output set point is chosen in *AOS*^*∞*^, then this set point would be operable, meaning it could be achieved with a bounded input sequence in *AIS*^*t*^ regardless of the disturbance sequences in *EDS*^*k*^. Since the disturbances in the *EDS* are Gaussian random variables, the operability condition could be relaxed by considering smaller ranges of disturbances realizations, and the *EDS* satisfaction percentage is the probability of achieving a stochastic set point after the relaxation. The stochastic operability concept is especially important when the manipulated inputs fail to compensate for the disturbance effects. In Figure 10b, the variance of the inlet methane concentration is increased from 2.5 to 5, and the *AOS*^*∞*^ associated with the expanded *EDS*^*k*^ is now empty. While the DMA-MR is not operable in this case, it is stochastically operable with an *EDS* satisfaction percentage of 85%. In other words, a set point in the small yellow-filled polytope in Figure 10b could be reached 85% of the time. This could potentially be used to help define chance constraints for stochastic model predictive controllers.

In the case study of the DMA-MR, all designs in the *DIS* have nonnegative measures of their *AOS*^*∞*^ with the *EDS*^*t*^ corresponding to the 99% highest density region. Therefore, the feasible design region is also the operable design region for this modular membrane reactor.

### 4.4 Closed-Loop Control Analysis of the DMA-MR

The controller considered for the closed-loop analysis in this subsection is a nonlinear model predictive control (NMPC). NMPC is formulated as a constrained nonlinear programming problem that optimizes a control objective while adopting the DMA-MR dynamic model as equality constraints. At every time step, the following optimization problem is solved:
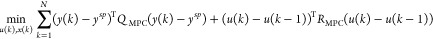
44subject to
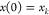
45
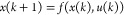
46
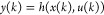
47
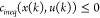
48in which the constraints in eqs 46–48 represent the dynamic model of the DMA-MR given in subsection 4.1, the predictive horizon *N* is chosen to be 10 minutes, the state-weighting matrix *Q*_MPC_ is a diagonal matrix with each weight equal to 100, the manipulated input suppression matrix *R*_MPC_ is an identity matrix, the set point *y*_*sp*_ is the steady-state outputs mapped from the operable design region, and the internal dynamic model of the NMPC is initialized by the current state *x*_*k*_. In practice, the current state *x*_*k*_ is calculated from the measurements by a state estimation layer. However, here the state estimator is not considered in the closed-loop performance analysis of the DMA-MR, and *x*_*k*_ is assumed to be directly measured.

**Figure 11:**
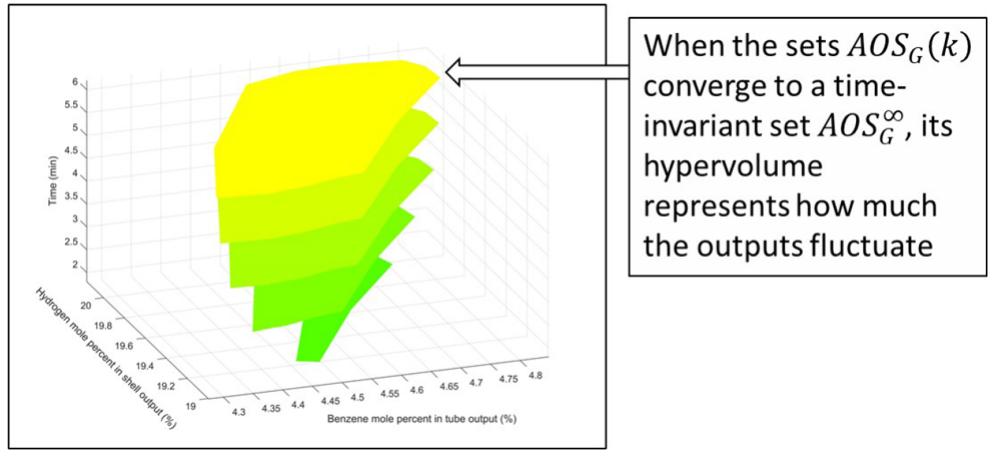
*AOS*_*G*_(*k*) of a DMA-MR with closed-loop NMPC.

The closed-loop analysis using the operability mapping proposed in subsection 3.4 is applied here for the DMA-MR. The control law *G*(*x*(*k*)) in eq 27 is chosen to be the above NMPC. The ranges of the process disturbances included in the *EDS*^*k*^ are assumed to be the same as the disturbance ranges in the dynamic operability analysis of subsection 3.3. For each operable design, the *AOS*_*G*_(*k*) is calculated until a time-invariant *AOS*_*G*_^*∞*^ is achieved with ε = 0.1 at the 8 minute mark. For an operable DMA-MR, the *AOS*_*G*_(*k*) is defined as the tightest polytopes that bound the fluctuations of the output concentrations under closed-loop NMPC. An example of the *AOS*_*G*_(*k*) obtained for the DMA-MR is provided in Figure 11. Since the feedback control law is fixed according to the state variables, the only expanding factors of the *AOS*_*G*_(*k*) for this case are the process disturbances. So the measure of the *AOS*_*G*_(*k*) represents the hypervolume of deviations from the set points caused by the disturbances.

**Figure 12:**
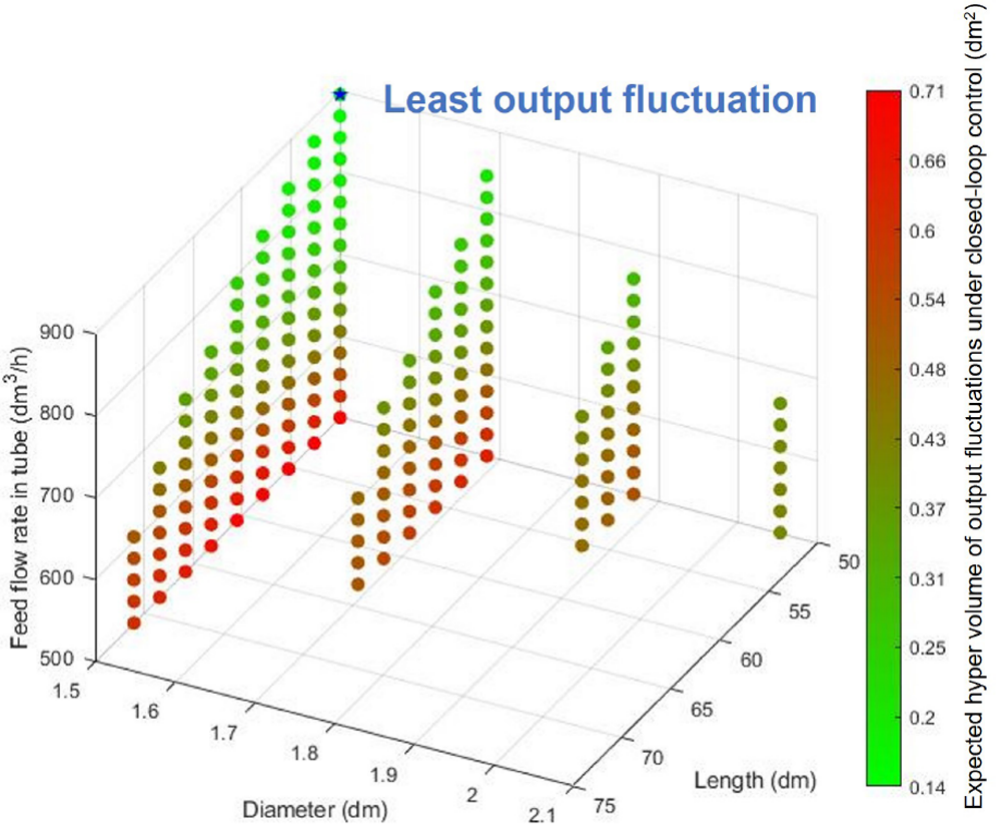
Closed-loop performance analysis with dynamic operability of the DMA-MR.

For each operable design, the closed-loop analysis is performed until the *AOS*_*G*_(*k*) reaches their respective time-invariant sets. The hypervolumes of different *AOS*_*G*_^*∞*^ sets for different operable designs are compared with each other to analyze their closed-loop performances. The summary of the measure μ of the *AOS*_*G*_^*∞*^ obtained for the DMA-MR is given in Figure 12. From this figure, the operable design coupled with an NMPC that gives the smallest set point deviations can be identified and shown as the point with the least output fluctuations.

## 5 Conclusions

In this article, the dynamic operability concepts are further extended starting from the classical operability concepts. The distinctions between steady-state operability and dynamic operability led to different implementations to find feasible and operable design regions. While steady-state and dynamic operability are inherent properties of the system and thus independent of the control laws, an adaptation for the operability measure and mapping is proposed to evaluate the closed-loop performance of different designs. The framework is applied to a modular DMA-MR for a gas processing application in the Marcellus Shale. In addition to the theoretical operability contributions provided, this work proposed a novel dynamic model of the DMA-MR and a control structure that guaranteed set point reachability regardless of the realizations of the disturbance. While dynamic operability is proven to be an effective tool for design and control assessment, the obtained dynamic mappings relied on a number of simulations that increased exponentially with the number of time steps. The main reason for the computational challenges when scaling the dynamic operability mapping procedure is the fact that each manipulated input can take a different value at a different moment in time. Thus, the longer the time horizon is in the dynamic operability mapping, the larger the number of input combinations is needed to generate all available input sequences. To address this issue, a state-space projection dynamic operability mapping is a subject of ongoing investigation.
